# The Pornography “Rebooting” Experience: A Qualitative Analysis of Abstinence Journals on an Online Pornography Abstinence Forum

**DOI:** 10.1007/s10508-020-01858-w

**Published:** 2021-01-05

**Authors:** David P. Fernandez, Daria J. Kuss, Mark D. Griffiths

**Affiliations:** Psychology Department, Nottingham Trent University, 50 Shakespeare Street, Nottingham, NG1 4FQ UK

**Keywords:** Pornography, Addiction, Sexual dysfunction, Abstinence, “Rebooting”, PornHub

## Abstract

A growing number of individuals using online forums are attempting to abstain from pornography (colloquially termed “rebooting”) due to self-perceived pornography-related problems. The present qualitative study explored phenomenological experiences of abstinence among members of an online “rebooting” forum. A total of 104 abstinence journals by male forum members were systematically analyzed using thematic analysis. A total of four themes (with a total of nine subthemes) emerged from the data: (1) abstinence is the solution to pornography-related problems, (2) sometimes abstinence seems impossible, (3) abstinence is achievable with the right resources, and (4) abstinence is rewarding if persisted with. Members’ primary reasons for initiating “rebooting” involved desiring to overcome a perceived addiction to pornography and/or alleviate perceived negative consequences attributed to pornography use, especially sexual difficulties. Successfully achieving and maintaining abstinence was typically experienced to be very challenging due to habitual behavior patterns and/or cravings triggered by a multiplicity of cues for pornography use, but a combination of internal (e.g., cognitive-behavioral strategies) and external (e.g., social support) resources made abstinence attainable for many members. A range of benefits attributed to abstinence by members suggest that abstaining from pornography could potentially be a beneficial intervention for problematic pornography use, although future prospective studies are needed to rule out possible third variable explanations for these perceived effects and to rigorously evaluate abstinence as an intervention. The present findings shed light on what the “rebooting” experience is like from members’ own perspectives and provide insights into abstinence as an approach for addressing problematic pornography use.

## Introduction

Pornography use is a common activity in the developed world, with nationally representative studies showing that 76% of men and 41% of women in Australia reported using pornography within the past year (Rissel et al., 2017), and that 47% of men and 16% of women in the U.S. reported using pornography at a monthly or greater frequency (Grubbs, Kraus & Perry, 2019a). *PornHub* (one of the largest pornography websites) reported in their annual review that they received 42 billion visits in 2019, with a daily average of 115 million visits a day (Pornhub.com, 2019).

### Problematic Pornography Use

Given the prevalence of pornography use, the potential negative psychological effects of pornography use have been the subject of increasing scientific attention in recent years. The available evidence generally indicates that although the majority of individuals who use pornography may do so without experiencing significant negative consequences, a subset of users may develop problems related to their pornography use (e.g., Bőthe, Tóth-Király, Potenza, Orosz, & Demetrovics, 2020; Vaillancourt-Morel et al., 2017).

One primary self-perceived problem related to pornography use concerns addiction-related symptomatology. These symptoms generally include impaired control, preoccupation, craving, use as a dysfunctional coping mechanism, withdrawal, tolerance, distress about use, functional impairment, and continued use despite negative consequences (e.g., Bőthe et al., 2018; Kor et al., 2014). Problematic pornography use (PPU) is most often conceptualized in the literature as a behavioral addiction despite “pornography addiction” not being formally recognized as a disorder (Fernandez & Griffiths, 2019). Nonetheless, the World Health Organization (WHO) recently included the diagnosis of compulsive sexual behavior disorder (CSBD) as an impulse control disorder in the eleventh revision of the *International Classification of Diseases* (ICD-11; World Health Organization, 2019), under which compulsive use of pornography might be subsumed. At the same time, it is important to note that research (Grubbs & Perry, 2019; Grubbs, Perry, Wilt, & Reid, 2019b) has shown that self-perceptions of being addicted to pornography might not necessarily reflect an actual addictive or compulsive pattern of pornography use. A model explaining pornography-related problems (Grubbs et al., 2019b) has suggested that although some individuals may experience a genuine pattern of impaired control in relation to their pornography use, other individuals may perceive themselves to be addicted to pornography due to moral incongruence (in the absence of a genuine pattern of impaired control). Moral incongruence occurs when an individual morally disapproves of pornography and yet engages in pornography use, resulting in a misalignment between their behavior and values (Grubbs & Perry, 2019). This incongruence might then lead to the pathologizing of their pornography use (Grubbs et al., 2019b). However, it should also be noted that this model does not rule out the possibility that both moral incongruence and genuine impaired control may be present simultaneously (Grubbs et al., 2019b; Kraus & Sweeney, 2019).

Research has also indicated that some pornography users might find their pornography use problematic due to perceived negative consequences attributed to their pornography use (Twohig, Crosby, & Cox, 2009). PPU has also been referred to in the literature as any use of pornography that creates interpersonal, vocational, or personal difficulties for the individual (Grubbs, Volk, Exline, & Pargament, 2015). Research on self-perceived adverse effects of pornography consumption has shown that some individuals report experiencing depression, emotional problems, decreased productivity, and damaged relationships as a result of their pornography use (Schneider, 2000). Although potential associations between pornography use and sexual dysfunctions are generally inconclusive (see Dwulit & Rzymski, 2019b), self-perceived negative effects on sexual functioning have also been reported by some pornography users, including erectile difficulties, decreased desire for partnered sexual activity, decreased sexual satisfaction, and reliance on pornographic fantasies during sex with a partner (e.g., Dwulit & Rzymski, 2019a; Kohut, Fisher, & Campbell, 2017; Sniewski & Farvid, 2020). Some researchers have used terms such as “pornography-induced erectile dysfunction” (PIED) and “pornography-induced abnormally low libido” to describe specific sexual difficulties attributed to excessive pornography use (Park et al., 2016).

### Abstinence from Pornography as an Intervention for Problematic Pornography Use

One common approach to addressing PPU involves attempting to completely abstain from viewing pornography. Most 12-step groups adapted for problematic sexual behaviors tend to advocate an abstinence approach to the specific type of sexual behavior that is problematic for the individual, including pornography use (Efrati & Gola, 2018). Within clinical interventions for PPU, abstinence is chosen by some pornography users as an intervention goal as an alternative to reduction/controlled use goals (e.g., Sniewski & Farvid, 2019; Twohig & Crosby, 2010).

Some limited prior research has suggested that there may be benefits to abstaining from pornography. Three studies that experimentally manipulated abstinence from pornography in non-clinical samples indicate that there may be some positive effects to short-term (2–3 weeks) abstinence from pornography (Fernandez, Kuss, & Griffiths, 2020), including greater relationship commitment (Lambert, Negash, Stillman, Olmstead, & Fincham, 2012), less delay discounting (i.e., showing preference for smaller and more immediate rewards rather than attaining larger but later rewards; Negash, Sheppard, Lambert, & Fincham, 2016), and insight into compulsive patterns in one’s own behavior (Fernandez, Tee, & Fernandez, 2017). There have also been a handful of clinical reports where pornography users were asked to abstain from pornography for relief of sexual dysfunctions attributed to their pornography use, including low sexual desire during partnered sex (Bronner & Ben-Zion, 2014), erectile dysfunction (Park et al., 2016; Porto, 2016), and difficulty achieving orgasm during partnered sex (Porto, 2016). In most of these cases, abstaining from pornography provided relief from their sexual dysfunction. Collectively, these findings provide some preliminary evidence that abstinence could potentially be a beneficial intervention for PPU.

### The “Rebooting” Movement

Notably, over the past decade, there has been a growing movement of pornography users utilizing online forums (e.g., *NoFap.com, r/NoFap,* *Reboot Nation*) attempting to abstain from pornography due to problems attributed to excessive pornography use (Wilson, 2014, 2016).^1^ “Rebooting” is a colloquial term used by these communities that refers to the process of abstaining from pornography (sometimes accompanied by abstaining from masturbation and/or having an orgasm for a period of time) in order to recover from the negative effects of pornography (Deem, 2014b; NoFap.com, n.d.). This process is called “rebooting” to connote imagery of the brain being restored to its original “factory settings” (i.e., before the negative effects of pornography; Deem, 2014b; NoFap.com, n.d.). Online forums dedicated to “rebooting” were founded as early as 2011 (e.g., r/NoFap, 2020) and membership on these forums have been growing rapidly since. For example, one of the largest English-language “rebooting” forums, the subreddit r/NoFap, had approximately 116,000 members in 2014 (Wilson, 2014), and this number has grown to more than 500,000 members as of 2020 (r/NoFap, 2020). However, what has yet to be adequately addressed within the empirical literature is what specific problems are driving an increasing number of pornography users on these forums to abstain from pornography in the first place, and what the pornography “rebooting” experience is like for these individuals.

Previous studies utilizing a diverse range of samples might provide some insight into the motivations and experiences of individuals who attempt abstinence from pornography and/or masturbation. In terms of motivations for abstinence, abstinence from pornography was shown to be driven by a desire for sexual purity in a qualitative study of Christian men (i.e., Diefendorf, 2015), while a qualitative study of Italian men on an online “pornography dependence” recovery forum showed that abstinence from pornography was motivated by perceptions of addiction and significant negative consequences attributed to pornography use, including impairment in social, occupational, and sexual functioning (Cavaglion, 2009). In terms of meanings associated with abstinence, a recent qualitative analysis of narratives of religious men’s pornography addiction recovery showed that they made use of both religion and science to make sense of their perceived addiction to pornography, and that abstinence from pornography for these men could be interpreted as an act of “redemptive masculinity” (Burke & Haltom, 2020, p. 26). In relation to coping strategies for maintaining abstinence from pornography, findings from three qualitative studies of men from different recovery contexts, the aforementioned Italian online forum members (Cavaglion, 2008), members of 12-step groups (Ševčíková, Blinka, & Soukalová, 2018), and Christian men (Perry, 2019), demonstrate that apart from making use of practical strategies, these individuals typically perceived that providing mutual support to each other within their respective support groups was instrumental to their ability to remain abstinent. A recent quantitative study of men from the subreddit r/EveryManShouldKnow (Zimmer & Imhoff, 2020) found that motivation to abstain from masturbation was positively predicted by perceived social impact of masturbation, perception of masturbation as unhealthy, decreased genital sensitivity, and one aspect of hypersexual behavior (i.e., dyscontrol). While useful, the findings from these studies are limited in their transferability to pornography users abstaining from pornography today as part of the “rebooting” movement because they are over a decade old, before the emergence of the movement (i.e., Cavalgion, 2008, 2009), because they were contextualized specifically within a 12-step recovery milieu (Ševčíková et al., 2018) or religious context (Burke & Haltom, 2020; Diefendorf, 2015; Perry, 2019), or because participants were recruited from a non-“rebooting” forum (Zimmer & Imhoff, 2020; see also Imhoff & Zimmer, 2020; Osadchiy, Vanmali, Shahinyan, Mills, & Eleswarapu, 2020).

There has been little systematic investigation of abstinence motivations and experiences among pornography users on online “rebooting” forums, apart from two recent studies. The first study (Vanmali, Osadchiy, Shahinyan, Mills, & Eleswarapu, 2020) used natural language processing methods to compare posts on the r/NoFap subreddit (a “rebooting” forum) that contained text related to PIED (*n* = 753) to posts that did not (*n* = 21,966). The authors found that although both PIED and non-PIED discussions featured themes related to various aspects of relationships, intimacy and motivation, only PIED discussions emphasized themes of anxiety and libido. Also, PIED posts contained fewer “discrepancy words,” suggesting “a more assured writing style” (Vanmali et al., 2020, p. 1). The findings of this study suggest that the anxieties and concerns of individuals on “rebooting” forums are unique depending on the specific self-perceived pornography-related problem, and that further research is needed to better understand the different motivations of individuals who use these forums. Second, Taylor and Jackson (2018) conducted a qualitative analysis of posts by members of the r/NoFap subreddit. However, their study’s aim was not to focus on members’ phenomenological experiences of abstinence, but to apply a critical lens using discourse analysis, to illustrate how some members employed “idealized discourses of innate masculinity and the need for “real sex” to justify their resistance to pornography use and masturbation” (Taylor & Jackson, 2018, p. 621). While such critical analyses provide useful insights into the underlying attitudes of some members of the forum, experiential qualitative analyses of members’ experiences that “give voice” to their own perspectives and meanings are also needed (Braun & Clarke, 2013, p. 20).

### The Present Study

Accordingly, we sought to fill this gap in the literature by conducting a qualitative analysis of phenomenological experiences of abstinence among members of an online “rebooting” forum. We analyzed a total of 104 abstinence journals by male members of a “rebooting” forum using thematic analysis, using three broad research questions to guide our analysis: (1) what are members’ motivations for abstaining from pornography? and (2) what is the abstinence experience like for members? and (3) how do they make sense of their experiences? Findings of the present study will be useful for researchers and clinicians to obtain a deeper understanding of (1) the specific problems which are driving an increasing number of members on “rebooting” forums to abstain from pornography, which can inform clinical conceptualization of PPU; and (2) what the “rebooting” experience is like for members, which can guide development of effective treatments for PPU and inform understanding of abstinence as an intervention for PPU.

## Method

### Subjects

We collected data from an online “rebooting” forum, *Reboot Nation* (Reboot Nation, 2020). *Reboot Nation* was founded in 2014, and at the time of data collection (July 2019), the forum had over 15,000 registered members. On the *Reboot Nation* homepage, there are links to informational videos and articles describing negative effects of pornography and recovery from these effects through “rebooting.” To become a registered member of the *Reboot Nation* forum, an individual needs to create a username and password and provide a valid email address. Registered members can then immediately start posting on the forum. The forum provides a platform for members to connect with each other and discuss recovery from pornography-related problems (e.g., sharing helpful information and strategies for “rebooting,” or asking for support). There are five sections on the forum categorized by topic: “porn addiction,” “porn induced erectile dysfunction/delayed ejaculation,” “partners of rebooters and addicts” (where partners of people with PPU can ask questions or share their experiences), “success stories” (where individuals who have successfully achieved long-term abstinence can share their journey retrospectively), and “journals” (which allows members to document their “rebooting” experiences using journals in real time).

### Measures and Procedure

Before beginning data collection, the first author engaged in a preliminary exploration of the “journals” section by reading posts from the first half of the year 2019 to become familiar with the structure and content of journals on the forum. Members start journals by creating a new thread and typically use their first post to talk about their background and abstinence goals. This thread then becomes their personal journal, which other members are free to view and comment on to provide encouragement and support. These journals are a source of rich and detailed accounts of members’ abstinence experiences, and how they perceive and make sense of their experiences. An advantage of collecting data in this unobtrusive way (i.e., using existing journals as data as opposed to actively approaching members on the forum to participate in a study) allowed for observation of members’ experiences naturalistically, without researcher influence (Holtz, Kronberger, & Wagner, 2012). To avoid excessive heterogeneity in our sample (Braun & Clarke, 2013), we chose to restrict our analysis to male forum members aged 18 years and above.^2^ Based on our initial exploration of the journals, we determined two inclusion criteria for journals to be selected for analysis. First, the content of the journal would need to be sufficiently rich and descriptive to be subject to qualitative analysis. Journals that elaborated on motivations for initiating abstinence and described in detail the range of their experiences (i.e., thoughts, perceptions, feelings, and behavior) during the abstinence attempt fulfilled this criterion. Second, the duration of the abstinence attempt described in the journal would need to last at least seven days, but no longer than 12 months. We decided on this period to account for both early abstinence experiences (< 3 months; Fernandez et al., 2020) and experiences following periods of sustained longer-term abstinence (> 3 months).^3^

At the time of data collection, there were a total of 6939 threads in the male journal section. The forum categorizes journals by age range (i.e., teens, 20s, 30s, 40s, and above). Since our primary aim was to identify common patterns of the abstinence experience, irrespective of age group, we set out to collect a similar number of journals across three age groups (18–29 years, 30–39 years, and ≥ 40 years). The first author selected journals from the years 2016–2018 at random and perused the content of the journal. If it met the two inclusion criteria, it was selected. Throughout this selection process, it was ensured that there were always a balanced number of journals from each age group. Whenever an individual journal was selected, it was read in full by the first author as part of the process of data familiarization (described later in the “data analysis” section). This process was continued systematically until it was determined that data saturation had been reached. We ended the data collection phase at this saturation point. A total of 326 threads were screened and 104 journals were selected that met the inclusion criteria (18–29 years [*N* = 34], 30–39 years [*N* = 35], and ≥ 40 years [*N* = 35]. The mean number of entries per journal was 16.67 (*SD* = 12.67), and the mean number of replies per journal was 9.50 (*SD* = 8.41). Demographic information and pertinent information about members (i.e., self-perceived addiction to pornography or other substances/behaviors, sexual difficulties, and mental health difficulties) were extracted from their journals wherever reported. Sample characteristics are summarized in Table 1. Of note, 80 members reported being addicted to pornography, while 49 members reported having some sexual difficulty. A total of 32 members reported both being addicted to pornography and having some sexual difficulty.

**Table 1:** Sample characteristics

	Frequency	%
Age group		
18–29 years	34	32.7
30–39 years	35	33.7
40 years and above	35	33.7
Marital status		
Single	11	10.6
Dating/in a relationship	10	9.6
Married	33	31.7
Divorced	1	1.0
Did not specify	49	47.1
Sexual orientation		
Heterosexual/straight	2	1.9
Bisexual	2	1.9
Gay	2	1.9
Did not specify	98	94.2
Religious affiliation		
Christian/Catholic	9	8.7
Muslim	1	1.0
Atheist	1	1.0
Did not specify	93	89.4
Self-perceived addiction to pornography		
Yes	80	76.9
No	4	3.8
Did not specify	20	19.2
Self-perceived sexual difficulty		
Erectile difficulties	42	40.4
Low desire for partnered sex	8	7.7
Delayed ejaculation	2	1.9
Not/none reported	55	52.9
Self-perceived addiction to pornography + sexual difficulty combinations		
Addiction reported but sexual difficulty not reported	48	46.1
Sexual difficulty reported but addiction not reported	17	16.3
Both addiction and sexual difficulty reported	32	30.8
Neither addiction nor sexual difficulty reported	7	6.7
Other self-perceived addictions/problematic behaviors		
Sex/sexting/sex chats	6	5.8
Internet/mobile phone use	2	1.9
Nicotine/tobacco	2	1.9
Alcohol	1	1.0
Not/none reported	93	89.4
Self-perceived mental health difficulties		
Depression	10	9.6
Social anxiety	5	4.8
Generalized anxiety	4	3.8
Suicidality	3	2.9
Attention-deficit/hyperactivity disorder	2	1.9
Obsessive compulsive disorder	2	1.9
Not/none reported	78	75.0

*N*=104. Age range=18–63 years

### Data Analysis

We analyzed the data using a phenomenologically informed thematic analysis (TA; Braun & Clarke, 2006, 2013). Thematic analysis is a theoretically flexible method which allows researchers to conduct a rich, detailed analysis of patterned meaning across a dataset. Given our phenomenological approach to data analysis, our goal was to “obtain detailed descriptions of an experience as understood by those who have that experience in order to discern its essence” (Coyle, 2015, p. 15)—in this case, the experience of “rebooting” as understood by members of a “rebooting” forum. We situated our analysis within a critical realist epistemological framework, which “affirms the existence of reality…but at the same time recognizes that its representations are characterized and mediated by culture, language, and political interests rooted in factors such as race, gender, or social class” (Ussher, 1999, p. 45). This means we took members’ accounts at face value and considered them to be generally accurate representations of the reality of their experiences, while acknowledging possible influences of the sociocultural context in which they occur. Therefore, in the present analysis, we identified themes at the semantic level (Braun & Clarke, 2006), prioritizing members’ own meanings and perceptions.

We used NVivo 12 software throughout the entire data analysis process and followed the process of data analysis outlined in Braun and Clarke (2006). First, journals were read by the first author upon selection and then re-read for data familiarization. Next, the entire dataset was systematically coded by the first author, in consultation with the second and third authors. Codes were derived using a bottom-up process, meaning that preconceived coding categories were not imposed upon the data. Data were coded at a basic semantic level (Braun & Clarke, 2013), resulting in 890 unique data-derived codes. These codes were then merged once patterns started emerging to form higher level categories. For example, the basic codes “honesty is liberating” and “accountability makes abstinence possible” were grouped into a new category, “accountability and honesty,” which was in turned grouped under “effective coping strategies and resources.” In addition, descriptive information from each journal pertaining to the abstinence attempt in general (i.e., goal of abstinence and inferred duration of the abstinence attempt) was also systematically extracted. Once the entire data set was coded, codes were reviewed and then added or modified as necessary to ensure consistent coding across the data set. Candidate themes were then generated from the codes by the first author, guided by the research questions of the study. Themes were refined after review by the second and third authors and finalized once a consensus was reached by all three of the research team.

### Ethical Considerations

The ethics committee of the research team’s university approved the study. From an ethical standpoint, it was important to consider whether the data were collected from an online venue considered to be a “public” space (British Psychological Society, 2017; Eysenbach & Till, 2001; Whitehead, 2007). The *Reboot Nation* forum is easily found using search engines, and posts on the forum are readily accessible for viewing to anyone without requiring registration or membership. Therefore, it was concluded that the forum was “public” in nature (Whitehead, 2007), and informed consent from individual members was not required (as did the authors’ university ethics committee). Nonetheless, to further protect the privacy and confidentiality of members of the forum, all usernames reported in the results have been anonymized.

## Results

To provide context for our analysis, a summary of abstinence attempt characteristics is provided in Table 2. In terms of abstinence goals, 43 members intended to abstain from pornography, masturbation, and orgasm, 47 members intended to abstain from pornography and masturbation, and 14 members intended to abstain from pornography. This means that a sizeable proportion of the sample (at least 86.5%) was intending to abstain from masturbation in addition to abstaining from pornography. However, at the outset of their abstinence attempt, almost all members did not specify an exact time frame for their abstinence goals or indicate whether they were intending to quit any of these behaviors forever. Therefore, we were unable to ascertain whether members were typically interested in abstaining temporarily or ceasing the behavior permanently. We inferred the total duration of abstinence attempt for each journal based on members’ explicit statements (e.g., “on day 49 of the reboot”), or in the absence of explicit statements, through deduction based on dates of members’ posts. The majority of inferred total durations of abstinence attempts were between seven and 30 days (52.0%), and the median inferred total duration of all abstinence attempts was 36.5 days. However, it is important to note that members did not necessarily stop attempting to abstain beyond these periods—these durations merely reflect the implied length of the abstinence attempt recorded in the journal. Members could have continued with the abstinence attempt, but stopped posting in their journals.

**Table 2:** Characteristics of abstinence attempts

	Frequency	%
Goal of abstinence (explicitly stated/implied)		
Abstinence from pornography, masturbation, and orgasm	43	41.3
Abstinence from pornography and masturbation (orgasm permitted/undecided/not mentioned)	47	45.2
Abstinence from pornography (masturbation and/or orgasm permitted/undecided/not mentioned)	14	13.5
Inferred duration of abstinence attempt		
7 days–30 days	54	52.0
1 month–3 months	22	21.2
3 months–6 months	13	12.5
6 months–1 year	15	14.4

Median duration of abstinence attempts = 36.5 days

A total of four themes with nine subthemes were identified from the data analysis (see Table 3). In the analysis, frequency counts or terms denoting frequency are sometimes reported. The term “some” refers to less than 50% of members, “many” refers to between 50% and 75% of members, and “most” refers to more than 75% of members.^4^ As a supplementary step, we used the “crosstab” function in NVivo12 to explore if there were any notable differences in frequency of abstinence experiences across the three age groups. These were subjected to chi-square analyses to determine if these differences were statistically significant (see Appendix A). Age-related differences are highlighted under their corresponding theme below.

**Table 3:** Themes derived from thematic analysis of the dataset

Themes	Subthemes
Abstinence is the solution to pornography-related problems	Abstinence motivated by negative effects attributed to pornography use
	Abstinence about “rewiring” the brain
	Abstinence as the only feasible way to recover
Sometimes abstinence seems impossible	Navigating sexuality during the “reboot”
	The inescapability of cues for pornography use
	The insidiousness of the relapse process
Abstinence is achievable with the right resources	External resources: Social support and barriers to pornography access
	Internal resources: An arsenal of cognitive-behavioral strategies
Abstinence is rewarding if persisted with	Regaining control
	An array of psychological, social, and sexual benefits

To elucidate each theme, a selection of illustrative quotes is provided, with accompanying member code (001-104) and age. Nonsignificant spelling errors have been corrected to aid readability of the extracts. In order to make sense of some of the language used by members, a brief explanation of commonly used acronyms is necessary. The acronym “PMO” (pornography/masturbation/orgasm) is often used by members to refer to the process of watching pornography while masturbating to orgasm (Deem, 2014a). Members often group these three behaviors together because of how frequently their pornography use is accompanied by masturbating to orgasm. When discussing these behaviors separately, members often acronymize watching pornography as “P,” masturbating as “M” and having an orgasm as “O.” Acronymizations of combinations of these behaviors are also common (e.g., “PM” refers to watching pornography and masturbating but not to the point of orgasm, and “MO” refers to masturbating to the point of orgasm without watching pornography). These acronyms are also sometimes used as a verb (e.g., “PMO-ing” or “MO-ing”).

### Abstinence Is the Solution to Pornography-Related Problems

Members’ initial decision to attempt “rebooting” was founded on the belief that abstinence is the logical solution for addressing pornography-related problems. Abstinence was initiated because there was the belief that their pornography use was leading to serious negative consequences in their lives—therefore, removing pornography use would alleviate these effects through “rewiring” the brain. Because of the perceived addictive nature of pornography use, a reduction/controlled use approach to the behavior was not viewed as a viable strategy for recovery.

#### Abstinence Motivated by Negative Effects Attributed to Pornography Use

Three main consequences attributed to excessive pornography use were cited by members as motivations for initiating abstinence. First, for many members (*n *= 73), abstinence was motivated by a desire to overcome a perceived addictive pattern of pornography use (e.g., *“*I’m 43 now and I’m addicted to porn. I think the moment to escape from this horrible addiction has arrived*”* [098, 43 years]). Accounts of addiction were characterized by the experience of compulsivity and loss of control (e.g., *“*I’m trying to stop but it is so hard I feel that there is something pushing me to porn*”* [005, 18 years]), desensitization and tolerance to the effects of pornography over time (e.g., *“*I don’t really feel anything anymore when watching porn. It is sad that even porn has become so unexciting and unstimulating*”* [045, 34 years]), and distressing feelings of frustration and disempowerment (*“*I hate that I don’t have the strength to JUST STOP…I hate that I have been powerless against porn and I want to regain and assert my power*”* [087, 42 years].

Second, for some members (*n *= 44), abstinence was motivated by a desire to relieve their sexual difficulties, based on the belief that these difficulties (erectile difficulties [*n *= 39]; diminished desire for partnered sex [*n *= 8]) were (possibly) pornography-induced. Some members believed that their problems with sexual functioning were a result of a conditioning of their sexual response predominantly to pornography-related content and activity (e.g., *“*I notice how I lacked enthusiasm for the body of the other…I have conditioned myself to enjoy sex with the laptop*”* [083, 45 years]). Of the 39 members who reported erectile difficulties as a reason for initiating abstinence, 31 were relatively certain that they were suffering from “pornography-induced erectile dysfunction” (PIED). Others (*n *= 8) were less certain of definitively labeling their erectile difficulties as being “pornography-induced” due to wanting to rule out other possible explanations (e.g., performance anxiety, age-related factors, etc.), but decided to initiate abstinence in case they were indeed pornography-related.

Third, for some members (*n *= 31), abstinence was motivated by a desire to alleviate perceived negative psychosocial consequences attributed to their pornography use. These perceived consequences included increased depression, anxiety and emotional numbness, and decreased energy, motivation, concentration, mental clarity, productivity, and ability to feel pleasure (e.g., *“*I know it has tremendous negative effects on my concentration, motivation, self-esteem, energy level*”* [050, 33 years].” Some members also perceived negative impacts of their pornography use on their social functioning. Some described a sense of decreased connection with others (e.g., “(PMO)…makes me less interested and friendly to people, more self-absorbed, gives me social anxiety and makes me just not care about anything really, other than staying home alone and jerking off to porn” [050, 33 years]), while others reported a deterioration of specific relationships with significant others and family members, especially romantic partners.

Notably, a small proportion of members (*n *= 11) reported that they morally disapproved of pornography in some way, but only a few of these (*n *= 4) explicitly cited moral disapproval as a reason for initiating “rebooting” (e.g., “I am leaving porn because this shit is disgusting. Girls are being raped and tortured and used as fuck objects in this shit” [008, 18 years]). However, for these members, moral incongruence was not listed as the only reason for initiating abstinence but was accompanied by one of the other three primary reasons for abstinence (i.e., perceived addiction, sexual difficulties, or negative psychosocial consequences).

#### Abstinence About “Rewiring” the Brain

Abstinence was approached by some members based on an understanding of how their pornography use might have been negatively impacting their brains. Abstinence was viewed as the logical solution to reversing the negative effects of pornography, as a process that would “rewire” the brain (e.g., “I know I have to abstain in order to let my pathways heal and settle my brain” [095, 40s]). The concept of neuroplasticity in particular was a source of hope and encouragement for some members, which led them to believe that the negative effects of pornography may be reversible through abstinence (e.g., “Brain plasticity is the real saving process that will rewire our brain” [036, 36 years]). Some members described learning about pornography’s negative effects and “rebooting” through informational resources by influential figures respected by the “rebooting” community, especially Gary Wilson, host of the website *yourbrainonporn.com*. Wilson’s (2014) book (e.g., “The book Your Brain on Porn by Gary Wilson…introduced me to the idea of a reboot, this forum and really explained some things I did not know” [061, 31 years]) and 2012 TEDx talk (TEDx Talks, 2012; e.g., “I watched THE GREAT PORN EXPERIMENT yesterday, very interesting and informative” [104, 52 years]) were resources that were most frequently cited by members as being particularly influential in shaping their beliefs about pornography’s negative effects on the brain and “rebooting” as the appropriate solution to reversing these effects.

#### Abstinence as the Only Feasible Way to Recover

For some members who reported being addicted to pornography, abstinence was seen as the only feasible way to recover, largely due to a belief that using any pornography during abstinence would likely trigger addiction-related circuitry in the brain and lead to craving and relapse. Consequently, trying to engage in moderation instead of abstaining completely was seen as an unviable strategy:

I need to completely stop watching porn and any explicit material for that matter because whenever I watch any nsfw [not safe for work] content a pathway is created in my brain and when I get urges my brain automatically forces me to watch porn. Therefore, quitting p and m cold turkey is the only way to recover from this shit.” (008, 18 years)

### Sometimes Abstinence Seems Impossible

The second theme illustrates possibly the most striking feature of members’ “rebooting” experiences—how difficult it was to actually successfully achieve and maintain abstinence. At times, abstinence was perceived to be so difficult that it seemed impossible to achieve, as described by one member:

I am back on Struggle St., after a whole bunch of relapses. I am not sure how to successfully quit, sometimes it seems impossible. (040, 30s)

Three main factors appeared to contribute to the difficulty in achieving abstinence: navigating sexuality during the “reboot,” the seeming inescapability of cues for pornography use, and the relapse process experienced as being cunning and insidious.

#### Navigating Sexuality During the “Reboot”

A difficult decision that members had to make at the outset of the abstinence process was regarding acceptable sexual activity during the “reboot”: should masturbation without pornography and/or having an orgasm through partnered sexual activity be allowed in the short-term? For many members, the long-term goal was not to eliminate sexual activity altogether, but to redefine and learn a new “healthy sexuality” (033, 25 years) without pornography. This would likely mean incorporating partnered sex (e.g., *“*What we want is healthy natural sex with our partner, right?” [062, 37 years]) and/or masturbation without pornography (e.g., “I am okay with old-fashioned MO. I think it is possible to manage that in a healthy way without the debilitating effects of porn addiction*”* [061, 31 years]). However, what needed more consideration was whether allowing these behaviors in the short-term would help or hinder progress with their abstinence from pornography. On the one hand, allowing these activities in the initial phases of abstinence was perceived by some members to be a potential threat to abstinence, primarily due to what they colloquially dubbed the “chaser effect.” The “chaser effect” refers to strong cravings to PMO that arise after sexual activity (Deem, 2014a). Some reported experiencing this effect after both masturbation (e.g., “I find the more I MO the more I crave it and porn” [050, 33 years]) and partnered sexual activity (e.g., “I have noticed that after sex with wife the urges are stronger afterwards” [043, 36 years]). For these members, this resulted in a decision to temporarily abstain from masturbation and/or partnered sex for a period. On the other hand, for other members, abstaining completely from sexual activity was reported to lead to a build-up of sexual desire and cravings for pornography. Therefore, for these members, having a sexual outlet during the “reboot” did not impede progress, but in fact aided their ability to abstain from pornography (e.g., “I am finding that if I knock one out when I feel especially horny, then I am less likely to start making up excuses to resort to porn” [061, 36 years]).

It is interesting to note that paradoxically, close to one-third of members reported that instead of experiencing increased sexual desire, they experienced diminished sexual desire during abstinence, which they called the “flatline.” The “flatline” is a term that members used to describe a significant decrease or loss of libido during abstinence (although some appeared to have a broader definition for this to also include accompanying low mood and a sense of disengagement in general: (e.g., “I feel like I’m probably in a flatline right now as the desire to engage in any sort of sexual activity is almost nonexistent” [056, 30s]). Not being certain about when sexual desire would return was disconcerting for some (e.g., “Well, if I cannot have a regular orgasm when I feel like, what’s the point in living?” [089, 42 years]). The temptation for these members was to turn to PMO to “test” whether they could still function sexually during a “flatline” (e.g., “Bad thing though is that I start to wonder whether everything is still working in the way it should in my pants” [068, 35 years]).

#### The Inescapability of Cues for Pornography Use

What also made abstaining from pornography particularly challenging for many members was the seeming inescapability of cues that triggered thoughts of pornography and/or cravings to use pornography. First, there were seemingly ubiquitous external cues for pornography use. The most common source of external triggers was electronic media (e.g., “Dating sites, Instagram, Facebook, movies/TV, YouTube, online ads all can trigger relapses for me” [050, 33 years]). The unpredictability of sexually arousing content appearing in a television show or one’s social media feed meant that casual browsing of the internet could be risky. Seeing sexually attractive people in real life was also a trigger for some members (e.g., “I also quit the gym I was going to today as there’s way too much to look at there via woman in them tight yoga pants” [072, 57 years]), which meant that viewing anything sexually arousing, whether online or offline, could potentially be triggering. Also, the fact that members often accessed pornography while alone in their bedroom meant that their default immediate environment was already a cue for pornography use (e.g., “just lying in bed when I wake up and have nothing to do is a serious trigger” [021, 24 years]).

Second, there were also numerous internal cues for pornography use (primarily negative affective states). Because members had previously often relied on pornography use to regulate negative affect, uncomfortable emotions appeared to have become a conditioned cue for pornography use. Some members reported that they experienced heightened negative affect during abstinence. Some interpreted these negative affective states during abstinence as being part of withdrawal. Negative affective or physical states that were interpreted as being (possible) “withdrawal symptoms” included depression, mood swings, anxiety, “brain fog,” fatigue, headache, insomnia, restlessness, loneliness, frustration, irritability, stress, and decreased motivation. Other members did not automatically attribute negative affect to withdrawal but accounted for other possible causes for the negative feelings, such as negative life events (e.g., “I find myself getting agitated very easily these past three days and I don’t know if it’s work frustration or withdrawal” [046, 30s]). Some members speculated that because they had previously been using pornography to numb negative emotional states, these emotions were being felt more strongly during abstinence (e.g., *“*Part of me wonders if these emotions are so strong because of the reboot*”* [032, 28 years]). Notably, those in the 18–29 years age range were more likely to report negative affect during abstinence compared to the other two age groups, and those 40 years and above were less likely to report “withdrawal-like” symptoms during abstinence compared to the other two age groups. Regardless of the source of these negative emotions (i.e., withdrawal, negative life events, or heightened preexisting emotional states), it appeared to be very challenging for members to cope with negative affect during abstinence without resorting to pornography to self-medicate these negative feelings.

#### The Insidiousness of the Relapse Process

More than half of the sample (*n *= 55) reported at least one lapse during their abstinence attempt. More members in the 18–29 year age group reported at least one relapse (*n *= 27) compared to the other two age groups: 30–39 years (*n *= 16) and 40 years and above (*n *= 12). Relapse typically resembled an insidious process that often caught members off guard and left them feeling distressed immediately after. There appeared to generally be two ways by which lapses tended to occur. The first was when craving to use pornography was triggered for various reasons. Although craving was sometimes manageable, at other times craving was so severe that it was experienced as overwhelming and uncontrollable. When craving was severe, some members reported that it was sometimes accompanied by cunning rationalizations for relapse, as if they were being tricked by the “addicted brain” into relapse:

I had incredible strong urges to watch porn, and I found myself arguing with my own brain on the tune of: “this could be last time…,” “come on, do you think that just a small peek would be so bad,” “just today, and from tomorrow I stop again,” “I have to stop this pain, and there is only one way how to do that”…so basically, in the afternoon I managed to work very little, and instead I fought the urges continuously. (089, 42 years)

The second way in which the insidiousness of the relapse process manifested was that, even in the absence of strong cravings, lapses sometimes seemed to “just happen” on “autopilot,” to a point where it sometimes felt like relapse was happening *to them* (e.g., *“*it’s like I’m in autopilot or somethin’. I just stood there watching myself from the outside, like I’m dead, like I have no control whatsoever*”* [034, 22 years]). This automaticity was also sometimes observed when members found themselves subconsciously searching for sexually stimulating material online (e.g., sexually arousing videos on *YouTube*) that did not technically qualify as “pornography” (often referred to by members as “porn substitutes”). Browsing these “porn substitutes” was often a gradual gateway to a lapse.

### Abstinence Is Achievable with the Right Resources

Despite abstinence being difficult, many members found that abstinence was achievable with the right resources. A combination of external and internal resources appeared to be key in enabling members to successfully achieve and maintain abstinence.

#### External Resources: Social Support and Barriers to Pornography Access

Social support was a key external resource for many members that was crucial for them in maintaining abstinence. Members described receiving helpful support from many different sources, including family, partners, friends, support groups (e.g., 12-step groups), and therapists. However, the online forum itself was the most commonly cited source of support for members. Reading other members’ journals (especially success stories) and receiving supportive messages on one’s own journal was a primary source of inspiration and encouragement for members (e.g., *“*Seeing other journals and other posts motivate me and make me feel like I’m not alone*”* [032, 28 years]). Some members solicited further support by requesting another forum member to be their accountability partner, although for other members, simply maintaining a journal on the forum was sufficient to feel an increased sense of accountability. Honest sharing and accountability were described by some members as being essential to their ability to maintain motivation to stay abstinent (e.g., *“*The public oath and the public commitment is what is different now. Accountability. That was the element missing in the last 30 years*”* [089, 42 years]).

Another common external resource employed by members during abstinence was barriers that acted as impediments to easy access of pornography use. Some members reported installing applications on their devices that blocked pornographic content. These applications were typically found to be limited because there were usually means of circumventing them, but they were useful for creating one extra barrier that could intervene in a moment of vulnerability (e.g., *“*I want to reinstall K9 web-blocker. I can bypass it, but it still serves as a reminder*”* [100, 40 years]). Other strategies included using one’s electronic devices only in less triggering environments (e.g., never using their laptop in the bedroom, only using their laptop at work), or restricting their use of electronic devices altogether (e.g., temporarily leaving their smartphone with a friend, giving up their smartphone for a non-smartphone mobile phone). In general, external barriers were seen by members to be useful but not sufficient for maintaining abstinence because it was unrealistic to completely avoid any access to electronic devices, and also because internal resources were needed as well.

#### Internal Resources: An Arsenal of Cognitive-Behavioral Strategies

Most members reported making use of various internal resources (i.e., cognitive and/or behavioral strategies) to aid their abstinence. Day-to-day behavioral strategies (e.g., exercising, meditating, socializing, keeping busy, going out more often, and having a healthier sleep routine) were incorporated as part of an overall lifestyle change to minimize the frequency of triggering situations and craving. Cognitive and/or behavioral strategies were amassed by members over the abstinence attempt, often through trial-and-error experimentation, to regulate emotional states that could potentially precipitate a lapse (i.e., momentary cravings and negative affect). A behavioral approach to emotion regulation involved engaging in an alternative non-harmful activity instead of giving into the temptation to use pornography. Some members reported that taking a shower was particularly effective at combating cravings (e.g., “Tonight I was feeling extremely horny. So I took a very cold shower at 10 pm in very cold weather and boom! The urges are gone*”* [008, 18 years]). Attempting to suppress thoughts of pornography was a common cognitive strategy used, but some members realized over time that thought suppression was counterproductive (e.g., *“*I think I need to find a different strategy than, ‘don’t think about PMO, don’t think about PMO, don’t think about PMO.’ That makes me crazy and gets me to thinking about PMO*”* [099, 46 years]). Other common cognitive strategies used by members included mindfulness-related techniques (e.g., accepting and “riding” the craving or negative emotion) and reframing their thinking. Writing in their journals as they were experiencing craving or immediately after a lapse appeared to provide a particularly useful avenue for members to engage in motivating self-talk and reframe unhelpful thinking.

### Abstinence Is Rewarding if Persisted With

Members who persisted with abstinence typically found it to be a rewarding experience, despite its difficulties. The pain of abstinence appeared to be worth it because of its perceived rewards, as described by one member: *“*It has not been an easy ride, but it has been totally worth it*”* (061, 31 years). Specific benefits described included an increased sense of control, as well as improvements in psychological, social, and sexual functioning.

#### Regaining Control

A major perceived benefit of abstinence described by some members revolved around regaining a sense of control over their pornography use and/or their lives in general. After a period of abstinence, these members reported decreased salience, craving, and/or compulsivity with regard to their pornography use:

My porn desires are way down and it is way easier to fight my urges. I find I hardly think about it at all now. I am so pleased that this reboot has had the effect on me I wanted so badly. (061, 31 years)

Successfully abstaining from pornography for a period of time was also reported to result in an increased sense of self-control over pornography use and pornography abstinence self-efficacy (e.g., *“*It seems I’ve developed good self-control to avoid pornographic material” [004, 18 years]). Some members felt that as a result of exercising self-control over their pornography use, this newfound sense of self-control extended to other areas of their lives as well.

#### An Array of Psychological, Social, and Sexual Benefits

Many members reported experiencing various positive cognitive-affective and/or physical effects that they attributed to abstinence. The most common positive effects related to improvements in day-to-day functioning, including improved mood, increased energy, mental clarity, focus, confidence, motivation, and productivity (e.g., *“*No porn, no masturbation and I had more energy, more mental clarity, more happiness, less tiredness*”* [024, 21 years]). Some members perceived that abstaining from pornography resulted in feeling less emotionally numb and in an ability to feel their emotions more intensely (e.g., *“*I just ‘feel’ on a deeper level. with work, friends, past times, there have been waves of emotions, good & bad, but it’s a great thing*”* [019, 26 years]). For some, this resulted in enhanced experiences and an increased ability to feel pleasure from ordinary day-to-day experiences (e.g., “My brain can get so much more excited about little things and things that aren’t pure pleasure…like socializing or writing a paper or playing sports*”* [024, 21 years]). Of note, more members in the 18–29 age group reported positive affective effects during abstinence (*n *= 16) compared to the other two age groups, 30–39 (*n *= 7) and ≥ 40 (*n *= 2).

Perceived positive effects of abstinence on social relationships were also reported. Increased sociability was reported by some members, while others described improved relationship quality and an increased sense of connection with others (e.g., *“*I am feeling closer to my wife than I have in a long time*”* [069, 30s]). Another common benefit attributed to abstinence centered on perceived improvements in sexual functioning. Some members reported an increase in desire for partnered sex, which represented a welcome shift away from only being interested in masturbating to pornography (e.g., *“*I was so horny but the good thing was that I was horny for sexual experience with another human being. Not interested in porn induced orgasm*”* [083, 45 years]). Increased sexual sensitivity and responsiveness were reported by some members. Of the 42 members who reported erectile difficulties at the outset of the abstinence attempt, half (*n *= 21) reported at least some improvements in erectile function after abstaining for a period of time. Some members reported partial return of erectile function (e.g., “It was only about a 60% erection, but what was important is that it was there” [076, 52 years]), while others reported a complete return of erectile function (e.g., “I had sex with my wife both Friday night and last night, and both times were 10/10 erections that lasted quite a long time” [069, 30 years]). Some members also reported that sex was more pleasurable and satisfying than before (e.g., “I had two times (Saturday and Wednesday) the best sex in four years” [062, 37 years]).

## Discussion

The present qualitative study explored phenomenological experiences of abstinence among members of an online pornography “rebooting” forum. Thematic analysis of abstinence journals on the forum yielded four main themes (with nine subthemes): (1) abstinence is the solution to pornography-related problems, (2) sometimes abstinence seems impossible, (3) abstinence is achievable with the right resources, and (4) abstinence is rewarding if persisted with. The key contribution of this analysis is that it sheds light on why members of “rebooting” forums engage in “rebooting” in the first place, and what the “rebooting” experience is like for members from their own perspectives.

### Motivations for “Rebooting”

First, our analysis sheds light on what motivates individuals to initiate “rebooting” in the first place. Abstaining from pornography was viewed as the logical solution to their problems (Theme 1) because it was perceived that their pornography use led to serious negative consequences in their lives. Three kinds of perceived negative consequences of pornography use were the most frequently cited reasons for “rebooting”: (1) perceived addiction (*n *= 73), (2) sexual difficulties believed to be (possibly) pornography-induced (*n *= 44), and (3) negative psychological and social consequences attributed to pornography use (*n *= 31). It is important to note that these motivations were not necessarily mutually exclusive. For instance, 32 members reported having both an addiction to pornography and a sexual difficulty. At the same time, this meant that there was a proportion of members (*n *= 17) reporting possible pornography-induced sexual difficulties without necessarily reporting an addiction to pornography.

Members believed that abstaining from pornography use was able to reverse the negative effects of pornography use on the brain, and this belief was built upon an assimilation of neuroscientific concepts, such as neuroplasticity. Although the use of neuroscientific language to make sense of pornography-related struggles is not unique, as has been shown in previous qualitative analyses with religious samples (Burke & Haltom, 2020; Perry, 2019), it may be particularly characteristic of the “rebooting” community, given a “rebooting” culture that has likely developed from (and been shaped by) the recent proliferation of online sites disseminating information about supposed negative effects of pornography on the brain (Taylor, 2019, 2020) especially by influential figures respected by those in the “rebooting” community (Hartmann, 2020). Therefore, members’ motivations to attempt a “reboot” as a remedy for PPU is also likely influenced by “rebooting” culture and norms that have developed as a result of a collective consciousness of (especially senior) fellow members’ experiences and views, and the influence of prominent figures who have impacted the “rebooting” movement.

Of note, moral incongruence (Grubbs & Perry, 2019) was a less frequently cited reason for “rebooting” in this sample (*n *= 4), which suggests that (in general) members on “rebooting” forums might have differing motivations for abstaining from pornography use compared to religious individuals who do so primarily for moral reasons (e.g., Diefendorf, 2015). Even so, the possibility that moral incongruence might influence decisions to abstain from pornography use cannot be ruled out without follow-up research explicitly asking members if they morally disapprove of pornography. Also, the present analysis suggests that some members on “rebooting” forums may decide to abstain from masturbation (cf. Imhoff & Zimmer, 2020) primarily for the practical reason of helping themselves stay abstinent from pornography use (because they perceive that masturbating during a “reboot” triggers pornography cravings), and not necessarily because of a belief in the intrinsic benefits of semen retention (e.g., “superpowers” such as self-confidence and sexual magnetism), which some researchers have observed to be central to NoFap ideology (Hartmann, 2020; Taylor & Jackson, 2018).

### The “Rebooting” Experience

Second, our analysis illustrates what the “rebooting” experience is like from members’ own perspectives—successfully achieving and maintaining abstinence from pornography is very difficult (Theme 2), but it is achievable if an individual is able to make use of the right combination of resources (Theme 3). If abstinence is persisted with, it can be rewarding and worth the effort (Theme 4).

Abstaining from pornography was perceived to be difficult largely due to the interaction of situational and environmental factors, and the manifestation of addiction-like phenomena (i.e., withdrawal-like symptoms, craving, and loss of control/relapse) during abstinence (Brand et al., 2019; Fernandez et al., 2020). More than half of members recorded at least one lapse during their abstinence attempt. Lapses were either the result of the force of habit (e.g., accessing pornography on “autopilot”), or were precipitated by intense cravings that felt overwhelming and difficult to resist. Three main factors contributed to the frequency and intensity of cravings experienced by members: (1) the ubiquity of external cues for pornography use (especially sexual visual cues or situational cues such as being alone in one’s room), (2) internal cues for pornography use (especially negative affect, which pornography had previously been used to self-medicate prior to the “reboot”), and (3) the “chaser effect”—cravings which were the result of any sexual activity engaged in during abstinence. More members in the youngest age group (18–29 years) reported experiencing negative affect and at least one lapse during abstinence compared to the other two age groups. One possible explanation for this finding is that because libido tends to be higher for this age group compared to the other two age groups (Beutel, Stöbel‐Richter, & Brähler, 2008), it may be more difficult to refrain from using pornography as a sexual outlet. Another possible explanation is that abstaining from pornography use becomes more difficult the earlier an individual engages in habitual pornography viewing due to a greater dependency on the behavior developing. This explanation tallies with recent findings that age of first exposure to pornography was significantly associated with self-perceived addiction to pornography (Dwulit & Rzymski, 2019b), although more research is required to delineate the possible association between age of first exposure to pornography and PPU.

Importantly, members’ experiences showed that abstinence, although difficult, is achievable with the right combination of internal and external resources. Members were generally resourceful in experimenting with different coping strategies and resources to prevent relapse. For the most part, members built wide repertoires of effective internal resources (i.e., cognitive-behavioral strategies) over the abstinence period. An advantage of this trial-and-error approach was that members were able to customize, through trial-and-error, a program of recovery that worked for them. However, one downside of trial-and-error experimentation is that it sometimes led to the employment of ineffective relapse prevention strategies. For example, attempting to suppress thoughts of pornography was a common internal strategy used to deal with intrusive thoughts of pornography and cravings for pornography. Thought suppression has been demonstrated to be a counterproductive thought control strategy because it leads to rebound effects, i.e., an increase of those suppressed thoughts (see Efrati, 2019; Wegner, Schneider, Carter, & White, 1987). The fact that this was a relatively common strategy suggests that many individuals attempting to abstain from pornography, especially outside of a professional treatment context, might unknowingly engage in ineffective strategies such as thought suppression, and would benefit from psychoeducation about how to effectively manage cravings during abstinence. This specific example (and the various challenges faced by members while “rebooting”) highlight the importance of empirically supported interventions being developed, refined, and disseminated by the field to assist individuals with PPU in effectively regulating their pornography use. Interventions teaching mindfulness-based skills, for example, appear particularly suited to addressing many of the challenges experienced by members (Van Gordon, Shonin, & Griffiths, 2016). Learning to non-judgmentally accept the experience of craving with curiosity instead of suppressing it could be an effective means of dealing with craving (Twohig & Crosby, 2010; Witkiewitz, Bowen, Douglas, & Hsu, 2013). Cultivating dispositional mindfulness could help reduce automatic pilot behaviors that lead to lapses (Witkiewitz et al., 2014). Engaging in mindful sexual activity (Blycker & Potenza, 2018; Hall, 2019; Van Gordon et al., 2016) may allow for conditioning of the sexual response beyond pornography-related cues so that sexual activity can be enjoyed without dependence on pornography and pornography-related fantasy (e.g., masturbating without needing to fantasize to memories of pornography).

In terms of external resources, implementing barriers to pornography access, such as blocking applications, was described to be somewhat useful. However, social support and accountability appeared to be the external resources that were most instrumental to members’ ability to sustain abstinence. This finding is in line with previous qualitative analyses comprising diverse samples (Cavaglion, 2008, Perry, 2019; Ševčíková et al., 2018) that have highlighted the crucial role of social support in aiding successful abstinence. The “rebooting” forum itself was arguably the most important resource utilized by members that enabled them to successfully maintain abstinence. Honestly sharing their experiences in their journals, reading other members’ journals, and receiving encouraging messages from other members appeared to provide a strong sense of social support and accountability despite the lack of face-to-face interaction. This suggests that authentic interaction on online forums could provide a potentially equally beneficial alternative to in-person support groups (e.g., 12-step groups). The anonymity afforded by these online forums may even be an advantage because it may be easier for individuals with stigmatizing or embarrassing problems to acknowledge their problems and receive support online as opposed to in-person (Putnam & Maheu, 2000). Constant accessibility of the forum ensured that members could post in their journals whenever the need arose. Ironically, the characteristics (accessibility, anonymity, and affordability; Cooper, 1998) that contributed to members’ problematic pornography use in the first place were the same characteristics that added to the therapeutic value of the forum and were now facilitating their recovery from these very problems (Griffiths, 2005).

Members who persisted with abstinence typically found abstinence to be a rewarding experience and reported a range of perceived benefits which they attributed to abstaining from pornography. Perceived effects resembling pornography abstinence self-efficacy (Kraus, Rosenberg, Martino, Nich, & Potenza, 2017) or an increased sense of self-control in general (Muraven, 2010) were described by some members after successful periods of abstinence. Perceived improvements in psychological and social functioning (e.g., improved mood, increased motivation, improved relationships) and sexual functioning (e.g., increased sexual sensitivity and improved erectile function) were also described.

### Abstinence as an Intervention for Problematic Pornography Use

The wide range of reported positive effects of abstinence by members suggest that abstaining from pornography could potentially be a beneficial intervention for PPU. However, whether each of these perceived benefits resulted specifically from the removal of pornography use itself cannot be clearly established without follow-up studies using prospective longitudinal and experimental designs. For example, other intervening factors during abstinence such as making positive lifestyle changes, receiving support on the forum, or exerting greater self-discipline in general could have contributed to positive psychological effects. Or, changes in psychological variables (e.g., reduction in depression or anxiety) and/or changes in sexual activity (e.g., reduction in masturbation frequency) during abstinence could have contributed to improvements in sexual functioning. Future randomized controlled studies isolating the effects of abstaining from pornography (Fernandez et al., 2020; Wilson, 2016) in particular are needed to validate whether each of these specific perceived benefits can be conclusively attributed to the removal of pornography use specifically, and to rule out possible third variable explanations for these perceived benefits. Also, the current study design allowed mainly for observation of perceived positive effects of abstinence, and less so for perceived negative effects. This is because it is likely that the sample overrepresents members who found abstinence and online forum interaction to be beneficial, and as such might have been more likely to persist with abstinence and continue posting in their journals. Members who found abstinence and/or online forum interaction to be unhelpful may have merely stopped posting in their journals instead of articulating their negative experiences and perceptions, and therefore may be underrepresented in our analysis. For abstinence (and “rebooting”) to be properly evaluated as an intervention for PPU, it is important to first examine whether there are any possible adverse or counterproductive consequences of abstinence as an intervention goal and/or approaching the abstinence goal in a specific way. For example, being overly preoccupied with the goal of avoiding pornography (or anything that could trigger thoughts and/or cravings for pornography) could paradoxically increase preoccupation with pornography (Borgogna & McDermott, 2018; Moss, Erskine, Albery, Allen, & Georgiou, 2015; Perry, 2019; Wegner, 1994), or attempting abstinence without learning effective coping skills for dealing with withdrawal, craving or lapses, could potentially do more harm than good (Fernandez et al., 2020). Future research investigating abstinence as an approach to PPU should account for potential adverse effects in addition to potential positive effects.

Finally, the fact that abstinence was perceived to be so difficult raises an important question for researchers and clinicians to consider—is complete abstinence from pornography always necessary to address PPU? It is noteworthy that there appeared to be little consideration among members for a reduction/controlled use approach to recovery from pornography-related problems (in lieu of an abstinence approach) because of the belief that controlled use is unachievable due to the addictive nature of pornography—which is reminiscent of the 12-step approach to addictive/compulsive pornography use (Efrati & Gola, 2018). It is worth noting that within clinical interventions for PPU, reduction/controlled use goals have been seen as a valid alternative to abstinence goals (e.g., Twohig & Crosby, 2010). Some researchers have recently raised concerns that abstinence might not be the most realistic intervention goal for some individuals with PPU, in part because of how arduous a task it may be perceived to be, and propose prioritizing goals such as self-acceptance and acceptance of pornography use over abstinence (see Sniewski & Farvid, 2019). Our findings do suggest that for individuals who are intrinsically motivated to stay completely abstinent from pornography, abstinence, although difficult, may be rewarding if persisted with. Furthermore, acceptance and abstinence need not be mutually exclusive goals—a pornography user can learn to be accepting of themselves and their situation while desiring to stay abstinent if a life without pornography is valued (Twohig & Crosby, 2010). However, if reduction/controlled use of pornography is achievable and able to produce similarly beneficial outcomes to abstinence, then abstinence might not be necessary in all cases. Future empirical research comparing abstinence versus reduction/controlled use intervention goals is needed to clearly elucidate the advantages and/or disadvantages of either approach to recovery from PPU, and under what conditions one might be preferable over the other (e.g., abstinence might result in better outcomes for more severe cases of PPU).

### Study Strengths and Limitations

Strengths of the present study included: (1) unobtrusive data collection that eliminated reactivity; (2) analysis of journals instead of purely retrospective accounts of abstinence that minimized recall bias; and (3) broad inclusion criteria including a range of age groups, abstinence attempt durations, and abstinence goals that allowed for mapping out of commonalities of the abstinence experience across these variables. However, the study also has limitations warrant acknowledgment. First, unobtrusive data collection meant that we could not ask members questions about their experiences; therefore, our analysis was limited to content that members chose to write about in their journals. Second, subjective evaluation of symptoms without the use of standardized measures limits reliability of members’ self-reports. For example, research has shown that answers to the question “Do you think you have erectile dysfunction?” do not always correspond to International Index of Erectile Function (IIEF-5; Rosen, Cappelleri, Smith, Lipsky, & Pena, 1999) scores (Wu et al., 2007).

### Conclusion

The present study provides insights into the phenomenological experiences of pornography users part of the “rebooting” movement who are attempting to abstain from pornography due to self-perceived pornography-related problems. Findings of the present study are useful for researchers and clinicians to obtain a deeper understanding of (1) the specific problems which are driving an increasing number of pornography users to abstain from pornography, which can inform clinical conceptualization of PPU, and (2) what the “rebooting” experience is like, which can guide development of effective interventions for PPU and inform understanding of abstinence as an intervention for PPU. However, any conclusions from our analysis should be drawn with caution because of the inherent limitations in the study methodology (i.e., qualitative analysis of secondary sources). Follow-up studies that actively recruit members of the “rebooting” community and employ structured survey/interview questions are needed to validate the findings of this analysis and to answer more specific research questions about the experience of abstaining from pornography as a means of recovery from PPU.

## Appendix

See Table 4.

**Table 4:** Notable differences in frequencies of reported experiences across age groups

		18–29 (*n* = 34)	30–39 (*n* = 35)	≥40 (*n* = 35)	Relevant pairwise comparisons*
		*n*	*n*	*n*	
Attempted complete abstinence from pornography, masturbation, and orgasm	Yes	17	18	8	18–29 vs. ≥ 40: χ^2^(1) = 5.50
	No	17	17	27	30–39 vs. ≥ 40: χ^2^(1) = 6.12
Reported negative affect during abstinence	Yes	18	2	4	18–29 vs 30-39: χ^2^(1) = 18.69
	No	16	33	31	18–29 vs ≥ 40: χ^2^(1) = 13.68
Reported “withdrawal-like” symptoms during abstinence	Yes	14	12	4	18–29 vs ≥ 40: χ^2^(1) = 7.92
	No	20	23	31	30–39 vs ≥ 40: χ^2^(1) = 5.19
Reported at least one relapse during abstinence attempt	Yes	27	16	12	18–29 vs 30–39: χ^2^(1) = 8.34
	No	7	19	23	18–29 vs ≥ 40: χ^2^(1) = 14.29
Reported positive affective effects of abstinence	Yes	16	7	2	18–29 vs 30-39: χ^2^(1) = 5.68
	No	18	28	33	18–29 vs ≥ 40: χ^2^(1) = 15.29

*All significant at *p* < .025 (Bonferroni-adjusted)

## Footnotes

1 Forums that have an “r/” prefix are known as “subreddits,” online communities on the social media website Reddit that are dedicated to a specific topic.

2 Although there is a dedicated section on the forum for female forum members, the large majority of journals were by male forum members. This disproportion in the ratio of male to female journals mirrors previous research showing that men report much higher rates of pornography use (e.g., Hald, 2006; Kvalem et al., 2014; Regnerus et al., 2016), PPU (e.g., Grubbs et al., 2019a; Kor et al., 2014), and treatment-seeking for PPU (Lewczuk, Szmyd, Skorko, & Gola, 2017) compared to women. Given past research reporting notable gender differences in predictors of treatment-seeking for PPU (e.g., amount of pornography use and religiousness were significant predictors of treatment-seeking for women, but not for men—Gola, Lewczuk, & Skorko, 2016; Lewczuk et al., 2017), there may likewise be important differences in abstinence motivations and experiences between males and females on “rebooting” forums.

3 We chose a 12-month cutoff point as it can be reasonably expected that most perceived effects of “rebooting” would be observable within the first year of the abstinence attempt. Journals describing very long-term abstinence attempts (> 12 months), due to how long and detailed they are, would require a separate investigation analyzing a smaller total number of journals, ideally with an idiographic approach to data analysis.

4 It is important to bear in mind that because members were not responding to a structured list of questions, it is not possible to determine whether the rest of the sample shared (or did not share) the same experience if they did not report it. Consequently, where frequency counts or terms denoting frequency are reported, they are best understood as the minimum proportion of members in the sample who reported an experience, but the actual number of individuals who had the experience could have been larger.
